# Lithocholic acid controls adaptive immune responses by inhibition of Th1 activation through the Vitamin D receptor

**DOI:** 10.1371/journal.pone.0176715

**Published:** 2017-05-11

**Authors:** Thijs W. H. Pols, Teresa Puchner, H. Inci Korkmaz, Mariska Vos, Maarten R. Soeters, Carlie J. M. de Vries

**Affiliations:** 1 Department of Medical Biochemistry, Academic Medical Center, University of Amsterdam, Amsterdam, The Netherlands; 2 Department of Endocrinology and Metabolism, Academic Medical Center, University of Amsterdam, Amsterdam, The Netherlands; Karolinska Institutet, SWEDEN

## Abstract

Bile acids are established signaling molecules next to their role in the intestinal emulsification and uptake of lipids. We here aimed to identify a potential interaction between bile acids and CD4^+^ Th cells, which are central in adaptive immune responses. We screened distinct bile acid species for their potency to affect T cell function. Primary human and mouse CD4^+^ Th cells as well as Jurkat T cells were used to gain insight into the mechanism underlying these effects. We found that unconjugated lithocholic acid (LCA) impedes Th1 activation as measured by *i*) decreased production of the Th1 cytokines IFNγ and TNFαα, *ii*) decreased expression of the Th1 genes *T-box protein expressed in T cells* (*T-bet)*, *Stat-1* and *Stat4*, and *iii*) decreased STAT1α/β phosphorylation. Importantly, we observed that LCA impairs Th1 activation at physiological relevant concentrations. Profiling of MAPK signaling pathways in Jurkat T cells uncovered an inhibition of ERK-1/2 phosphorylation upon LCA exposure, which could provide an explanation for the impaired Th1 activation. LCA induces these effects via Vitamin D receptor (VDR) signaling since VDR RNA silencing abrogated these effects. These data reveal for the first time that LCA controls adaptive immunity via inhibition of Th1 activation. Many factors influence LCA levels, including bile acid-based drugs and gut microbiota. Our data may suggest that these factors also impact on adaptive immunity via a yet unrecognized LCA-Th cell axis.

## Introduction

Bile acids are synthesized in the liver and play crucial roles in the emulsification and uptake of lipids in the intestines [1]. The enzyme Cholesterol 7 α-hydroxylase (CYP7A1) is a key enzyme in bile acid synthesis and converts cholesterol to the bile acid-precursor 7α-hydroxycholesterol. Subsequent conversions and conjugation results in the generation of the primary bile acids, chenodeoxycholic acid (CDCA) and cholic acid (CA) [1]. In humans, cholecystokinin induces postprandial bile acid release from the liver into the intestine where bile acids facilitate the uptake of lipids. Glycine- and taurine-conjugated bile acids enter the gut where the microbiota deconjugate and metabolize primary bile acids to secondary bile acids lithocholic acid (LCA) and deoxycholic acid (DCA). Hereafter bile acids are re-absorbed and transported back to the liver via the portal vein for re-use. Bile acids cycle many times from the liver to the intestine, a process called the entero-hepatic cycle [1]. The synthesis and enterohepatic circulation of bile acids is mainly regulated by the Farnesoid X receptor (FXR), a nuclear bile acid receptor [1]. Outside the enterohepatic circulation, bile acids reach micromolar concentrations in the systemic circulation that fluctuate upon meal intake [2, 3].

The presence of bile acids in the systemic circulation and the fact that bile acids are able to induce specific signaling via dedicated bile acid receptors has established bile acids as hormone-like signaling molecules at the crossroad between food sensing and metabolism [4]. The hormone-like functions of bile acids comprise amongst others the induction of postprandial thermogenesis and Glucagon-like peptide (GLP)-1 release to improve glucose homeostasis, which are both mediated via the Takeda G-coupled receptor 5 (TGR5) [5–8]. Bile acid signaling is also shown to dampen innate immune responses via activation of TGR5 [6, 9].

Adaptive immune responses play crucial roles in almost all inflammatory processes, ranging from defense against pathogens to autoimmune diseases. A critical component of adaptive immunity are Th cells that are central in orchestrating immune responses [10]. A number of studies have revealed that plasma bile acid concentrations are, either positively or negatively, correlated to obesity, atherosclerosis and diabetes [11, 12]. Since adaptive immunity and Th cells play a role in these diseases [13, 14], we aimed to identify a potential direct link between bile acids and Th cells.

In the current study, we screened the most common bile acid species for their ability to affect Th cell inflammation. This approach led to the finding that unconjugated LCA inhibits Th1 activation in Jurkat T cells, human and mouse primary CD4^+^ Th cells. Upon deciphering the mechanism, we observed that LCA inhibits Th1 activation largely via the VDR. The VDR is well-studied in T cells and known to restrain Th1 inflammation [15–18]. Our findings unveil a yet unrecognized bile acid-adaptive immunity axis that potentially has major implications for drug and food strategies that impact on bile acid metabolism.

## Materials and methods

### CD4^+^ Th cell isolation and cell culture

Primary human CD4^+^ Th cells were purified from peripheral blood mononuclear cells using the CD4 T-cell isolation kit II (Miltenyi Biotec, California) together with LS columns (Miltenyi Biotec). Peripheral blood mononuclear cells were obtained as described previously [19]. We reached CD4^+^ Th cells purities between 95–98%. The cells were cultured in IMDM Medium (Gibco, United Kingdom) with 10% Fetal calf serum (FCS; Gibco) and 1% Penicillin/Streptomycin (Gibco) in the presence of recombinant human IL-2 (BioLegend, San Diego, California) at a concentration of 10 ng/ml. Primary human CD4^+^ Th cells were stimulated using CD3/CD28/CD137-coated Dynabeads T-activator (Life technologies, California) according to manufacturer’s instruction. Cells were typically stimulated with Dynabeads T-activator for 6 hours, unless stated otherwise.

Mouse spleens were harvested from C57Bl/6 mice after the animals were euthanized, which was approved by the animal experimentation committee of the Academic Medical Center. Primary mouse CD4^+^ Th cells were isolated by mashing mouse spleens through a 70-μm cellstrainer (BD Falcon, The Netherlands). We then incubated the cells with Red blood cell lysing buffer (Sigma-Aldrich, The Netherlands) for 10 minutes after which the splenocytes were washed. Using the mouse L3T4 CD4^+^ column kit (Miltenyi Biotec) together with MS colums (Miltenyi Biotec) we then isolated mouse primary CD4^+^ Th cells, with purities between 90–95%. Primary mouse CD4^+^ Th were cultured in RPMI-1640 medium (Gibco) in the presence of 1% Penicillin/Streptomycin (Gibco), 10% FCS and 10 ng/ml recombinant mouse IL-2 (Biolegend, California).

Jurkat T cells (Clone E6-1, ATCC, Germany) were cultured in RPMI-1640 medium (Gibco) in the presence of 1% Penicillin/Streptomycin (Gibco) and 10% FCS (Gibco). Phorbol myristate acetate (PMA; Sigma-Aldrich) and Ionomycin (Sigma-Aldrich) to activate T cells were used at concentrations of 100 ng/ml and 1 μg/ml, respectively. Cells were typically stimulated with PMA/Ionomycin (P/I) for 6 hours, unless stated otherwise. Staurosporine (Sigma-Aldrich) was used at a final concentration of 0.25 μM. Sodium taurodeoxycholate hydrate, taurocholic acid sodium salt hydrate, sodium taurochenodeoxycholate, sodium taurolithocholate, lithocholic acid, deoxycholic acid, chenodeoxycholic acid and cholic acid (all from Sigma-Aldrich) were dissolved in dimethylsulfoxide (DMSO) and subsequently further diluted in the culture medium to a final DMSO concentration of 0.1%. Cells were typically (pre)treated with bile acids for 24 hours, unless stated otherwise.

### Western blotting

For western blotting, cells were resuspended in lysis buffer containing protease inhibitor cocktail (Roche, Germany) and PhosSTOP (Roche). Gel elecrophoresis was performed using 10% polyacrylamide gels, and blotting was performed using the Trans-blot Turbo Transfer System (Bio-rad, The Netherlands) and the Trans-blot turbo RTA Transfer kit (LF PVDF, Bio-rad). Antibodies used were the following: ERK1/2 (137F5; 4695; Cell Signaling, The Netherlands), P-ERK1/2 (E-4;SC-7383; Santa Cruz Biotechnology, Texas), JNK1/2 (9252; Cell Signaling), P-JNK1/2 (81E11; 4668; Cell Signaling), P38 (9212; Cell Signaling), P-P38 (9211; Cell Signaling), P-STAT1 (Tyr-701; 9167; Cell Signaling), α-Tubulin (Cedarlane, Canada) and PARP1 (4C10-5, BD Biosciences, The Netherlands). As secondary antibodies we used Goat-anti Rabbit IRDye 800CW/680RD (LI-COR Biosciences, Nebraska), Goat-anti-mouse IRDye 800CW/680RD (LI-COR Biosciences). Imaging was performed on the Odyssey (LI-COR Biosciences), and quantification of signal intensity was done using Image Studio (LI-COR Biosciences). The signal intensity of phosphoproteins was corrected for the signal intensity of the (total) unphosphorylated protein (ERK/P38/JNK) or for the signal intensity of a housekeeping gene (α-Tubulin to correct for STAT1α/β).

### Real time RT-PCR

Messenger RNA was isolated using Aureum RNA isolation kits (Bio-rad), after which cDNA synthesis was made using IScript kits (Bio-rad). Real-time RT-PCR analysis was performed using the Lightcycler 480 (Roche). Data analysis was done using LC480 conversion software and LinReg software [20]. All mRNA expression levels were corrected for expression of the housekeeping genes *Ribosomal Protein P0 (P0)* and *β2 microglobulin (B2M)*. Used primer sequences were designed or taken from the Harvard primer bank [21], and can be found in the S1 Table.

### Determination of protein TNFα levels and flowcytometry

To analyze secreted TNFα protein levels, supernatant of cultured cells was stored at -80 degrees Celsius until analysis. Levels of TNFα were determined using the Bead Array Assay (BD Biosciences). Flowcytometry analysis was performed using FACSCalibur (BD Biosiences). The following flowcytometry antibodies were used: anti-mouse CD3e-APC (145-2C11, EBioscience, California), anti-mouse CD4-PeCyanine7 (GK1.5; EBioscience) and anti-mouse IFNγ-APC (XMG1.2; EBioscience, California). 7-Aminoactinomycin D (7-AAD; EBioscience) was used to mark dead cells. For intracellular IFNγ stainings, cells were permeablized using the Cytofix/Cytoperm kit (BD Biosciences). Analysis was done using FlowJo software (FlowJo Enterprise, Oregon).

### Transfections and luciferase assays

Transfections were performed by electroporation using the Amaxa apparatus (Lonza, Switzerland) and Cell Line Nucleofector Kit V (Lonza). The following constructs were transfected in Jurkat T cells: human TGR5 overexpressing plasmid (8), GFP plasmid (PMAX-GFP, Lonza) and CREB-luciferase reporter plasmid (Stratagene, California). Furthermore, we used human Smartpool VDR silencing RNA and control silencing RNA (Dharmacon, Colorado). Luciferase assays were performed using the Dual Luciferase Reporter Assay System (Promega, Wisconsin) kit in the Glomax Multi Detection System (Promega) apparatus. A plasmid encoding renilla luciferase was cotransfected and used as a control for transfection efficiency.

### Statistical analysis

The Student's t test was used to calculate the statistical significance. p<0.05 was considered statistically significant. Results represent the mean ± SEM.

## Results

### Bile acid screening in Jurkat T cells identifies LCA to inhibit IFNγ gene expression

**Fig 1:**
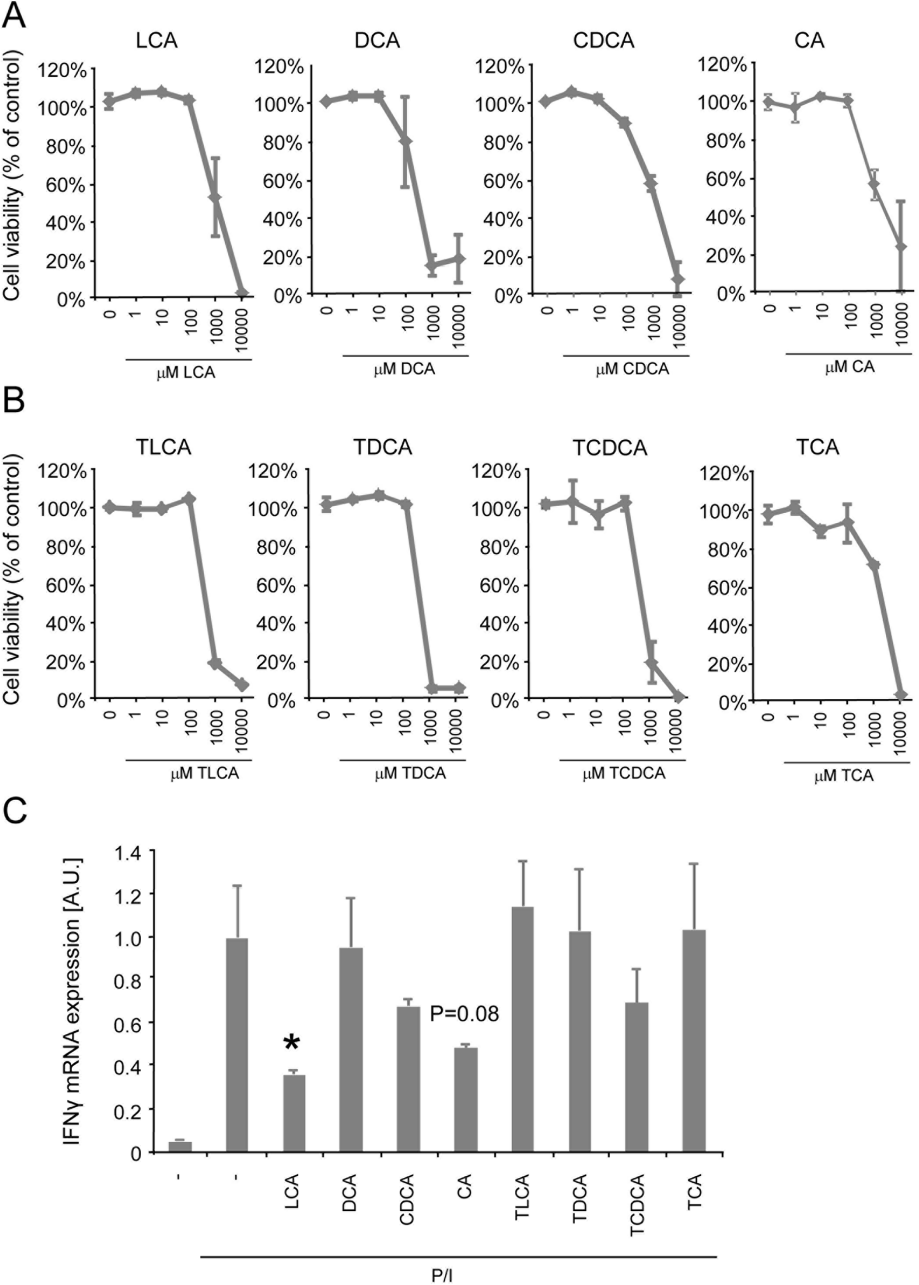
LCA inhibits IFNγ gene expression in Jurkat T cells **(A)** Cell viability of Jurkat T cells measured by flowcytometry analysis and expressed as percentage of control in response to incubation with unconjugated bile acids and **(B)** taurine-conjugated bile acids for 24 hours. **(C)** IFNγ mRNA expression of PMA/Ionomycin (P/I)-activated Jurkat T cells treated for 24 hours with 10 μM of different bile acid species. LCA, lithocholic acid; CDCA, chenodeoxycholic acid; CA, cholic acid; DCA, deoxycholic acid; TLCA, taurolithocholic acid; TDCA, taurodeoxycholic acid; TCDCA, taurochenodeoxycholic acid; TCA, taurocholic acid; AU, Arbitrary units. Results represent the mean ± SEM. *Statistically significant, P<0.05. Experiments were performed in triplicates and repeated at least twice.

Bile acids are currently appreciated as hormone-like signaling molecules having key roles beyond the emulsification of lipids in the intestines [4]. We set out to determine whether bile acids are capable of directly regulating adaptive immune responses. For this purpose, we screened a selection of the most common bile acid species for their ability to inhibit IFNγ production in activated Jurkat T cells, a human lymphoblast/T cell like cell line. The bile acids used for screening included taurine-conjugated and unconjugated variants of lithocholic acid (LCA), deoxycholic acid (DCA), chenodeoxcycholic acid (CDCA) and cholic acid (CA). First it was investigated whether the bile acids were not toxic to T cells, as bile acids are often associated with cytotoxicity [22]. We observed that bile acid treatment only induces cytotoxicity at high concentrations of 100 μM or above (Fig 1A and 1B). To exclude the possibility that bile acid treatment induces apoptosis that could affect Jurkat T cell activation, Poly ADP ribose polymerase (PARP1) cleavage was investigated, which is a readout of Caspase-3 activity and an early apoptosis marker. Staurosporine used as positive control induces clear PARP1 cleavage (panel A and B of S1 File). We observed that bile acids, predominantly the unconjugated bile acids, only induce clear PARP1 cleavage at very high concentrations of 1 mM (Panel A and B of S1 File).

We then performed our screening experiment in which *Interferon (IFN)-*γ mRNA expression was determined in Jurkat T cells in response to 10 μM bile acid, a concentration that does not affect cell viability. Analysis of *IFN*γ mRNA expression revealed that phorbol 12-myristate 13-acetate (PMA)/ionomycin treatment successfully activates Jurkat T cells. We observed that CA showed a trend (p = 0.08) towards decreased *IFN*γ mRNA expression. Interestingly, out of all conjugated and unconjugated bile acid species tested, we identified unconjugated LCA as the only bile acid species that significantly inhibited *IFN*γ mRNA expression (Fig 1C). In agreement with the findings on IFNγ, we observed that LCA also substantially inhibited IL-2 gene expression upon screening several bile acid species (Panel C of S1 File).

### LCA inhibits activation of CD4^+^ Th cells

**Fig 2:**
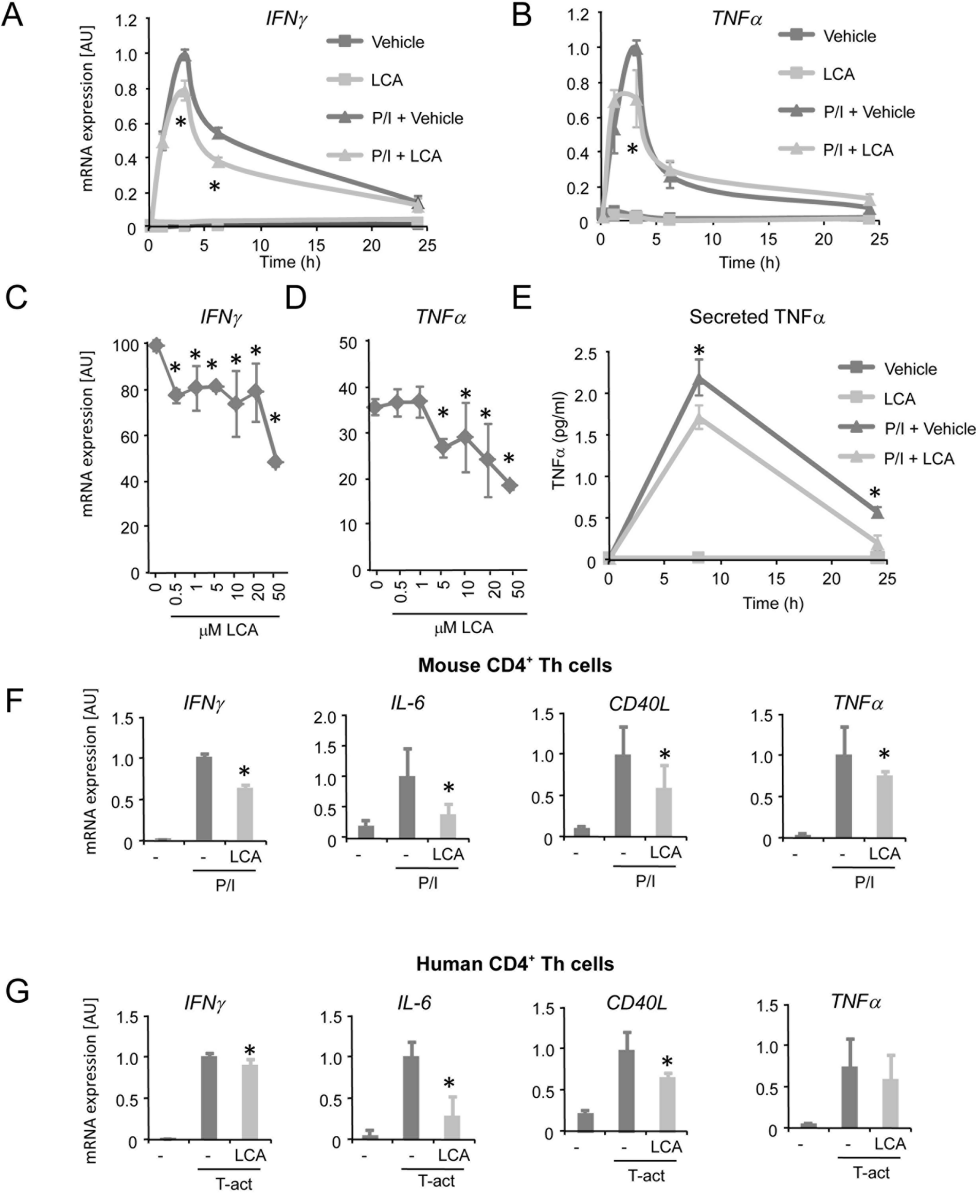
LCA inhibits CD4^+^ Th cell activation. **(A)** *IFN*γ mRNA expression and **(B)** *TNF*α mRNA expression in Jurkat T cells in response to 10 μM LCA treatment (light grey lines) or vehicle (dark grey lines) with P/I activation (triangles) or in resting Jurkat T cells (squares). **(C)** *IFN*γ and **(D)** *TNF*α mRNA expression in P/I-activated Jurkat T cells in response to increasing concentrations of LCA. **(E)** Secreted TNFα protein levels of Jurkat T cells in the supernatant in response to 10 μM LCA treatment (light grey lines) or vehicle (dark grey lines) with P/I activation (triangles) or in resting Jurkat T cells (squares). **(F)** mRNA expression of *IFN*γ, *IL-6*, *CD40L*, *TNF*α in primary mouse CD4^+^ Th cells activated with P/I and treated with 10 μμM LCA (light grey bars) or vehicle (dark grey bars). **(G)** mRNA expression of *IFN*γ, *IL-6*, *CD40L*, *TNF*α in primary human CD4^+^ Th cells stimulated with Dynabeads T-activator (T-act) and treated with 10 μM LCA (light grey bars) or vehicle (dark grey bars). P/I, PMA/ionomycin; LCA, lithocholic acid; AU, Arbitrary units. Results represent the mean ± SEM. *Statistically significant, P<0.05. Experiments were performed in triplicates and repeated at least twice.

To further characterize the inhibitory action of LCA on T cell activation, mRNA expression of the cytokines *Tumor necrosis factor* (*TNF*)*-*α, *Interleukin (IL)-8* and *CD40 Ligand* (*CD40L*) was analyzed. We observed decreased expression of the latter genes by LCA, which confirmed inhibition of Jurkat T cell activation (Panel D of S1 File). We then analyzed the effect of LCA on Jurkat T cell activation in a time course experiment in which we monitored *TNF*α and *IFN*γ mRNA expression in response to PMA/ionomycin stimulation. LCA treatment decreases *IFN*γ mRNA expression 3 and 6 hours after activation (Fig 2A). *TNF*α mRNA expression is significantly lower 3 hours after activation in response to LCA treatment (Fig 2B).

To gain insight into the concentrations necessary for LCA to mediate inhibition of IFNγ production, Jurkat T cells were treated with increasing concentrations of LCA. The lowest concentration of LCA tested (0.5 μM), is already sufficient to inhibit *IFN*γ mRNA expression in Jurkat T cells (Fig 2C). Additionally, *TNF*α (Fig 2D) *IL-2* and *IL-8* mRNA expression (Panel D of S1 File) is also dose-dependently decreased by LCA treatment. We assessed TNFα protein levels and observed decreased protein levels in the supernatant of LCA-treated Jurkat T cells (Fig 2E), which confirmed that the inhibition of cytokine mRNA expression translated to inhibition of protein secretion.

We next analyzed the capability of LCA to inhibit activation of primary mouse CD4^+^ Th cells. Mouse CD4^+^ Th cells were isolated from spleen, and flow cytometric analysis showed high purity of CD4^+^ Th cells (Panel F of S1 File). LCA treatment of P/I-stimulated primary mouse CD4^+^ Th cells resulted in decreased mRNA expression of *IFN*γ, *IL-6*, *CD40L*, *TNF*α (Fig 2F), *Chemokine (C-C motif) ligand (CCL)-1* and *IL-2* (Panel G of S1 File), reflecting decreased mouse CD4^+^ Th cell activation in response to LCA treatment. We furthermore performed intracellular stainings to detect IFNγ in CD3/CD28-activated primary mouse T helper cells. We found that LCA decreased the number of CD3/CD28-induced IFNγ positive cells, thereby confirming our findings in mouse T helper cells at protein level (Panel H of S1 File). LCA also inhibits the activation of human CD4^+^ Th cell activation as analyzed by decreased mRNA expression of *IFN*γ, *IL-6*, *CD40L* and *TNF*α (Fig 2G), as well as by *IL-1β* and *IL-2* (Panel I of S1 File). Taken together, our results demonstrate that LCA inhibits pro-inflammatory responses of Jurkat T cells, primary mouse CD4^+^ Th cells and primary human CD4^+^ Th cells.

### Inhibition of ERK phosphorylation by LCA

**Fig 3:**
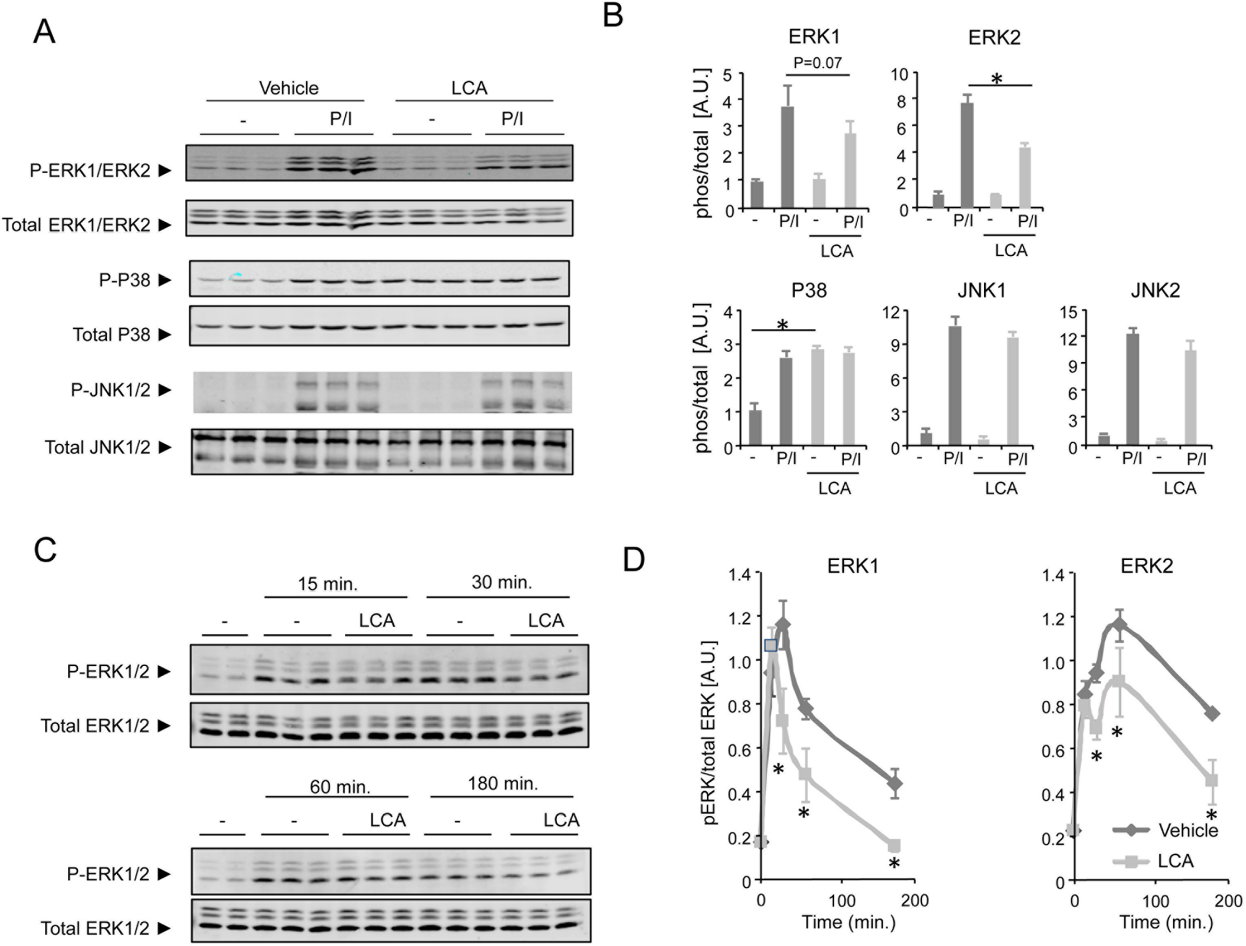
LCA inhibits ERK phosphorylation in Jurkat T cells. **(A)** Western blot images and **(B)** Quantification results of total and phosphorylated levels of ERK1/2, P38 and JNK1/2 in response to 10 μM LCA (light grey bars) or vehicle treatment (dark grey bars) in P/I-activated or resting Jurkat T cells. **(C)** Western blot images and **(D)** Quantification results of ERK1/2 phosphorylation in response to 10 μM LCA (light grey lines) or vehicle (dark grey lines) treatment in P/I-activated Jurkat T cells. LCA, lithocholic acid; P/I, PMA/ionomycin; AU, Arbitrary units. Results represent the mean ± SEM. *Statistically significant, P<0.05. Experiments were performed in triplicates and repeated at least twice.

To gain insight into the modulatory pathways that are responsible for the decreased CD4^+^ Th cell activation by LCA, we investigated the activation status of Mitogen-activated protein kinases (MAPK), Extracellular signal-regulated kinase (ERK)-1/2, c-Jun N-terminal kinase (JNK)-1/2 and P38 mitogen-activated protein kinase (P38) that are crucial in CD4^+^ Th cell activation [23]. PMA/ionomcyin treatment of Jurkat T cells induces clear phosphorylation of all MAPK tested, notably ERK1/2, JNK1/2 and P38 (Fig 3A and 3B). Of note, LCA increased basal P38 phosphorylation in the absence of PMA/ionomycin stimulation (Fig 3A and 3B). We also observed a strong inhibition of PMA/ionomycin-induced ERK1/2 phosphorylation by LCA (Fig 3A). Upon quantification of ERK phosphorylation levels, LCA tended to inhibit ERK1 phosphorylation (p = 0.07), and clearly inhibits ERK2 phosphorylation (Fig 3B). These inhibitory effects of LCA on PMA/ionomycin-induced phosphorylation are restricted to ERK, as we did not detect any changes in PMA/ionomycin-induced phosphorylation levels of JNK1/2 or P38 in response to LCA (Fig 3A and 3B).

None of the other bile acid species substantially affected MAPK signaling (S2 File), which is in agreement with our finding that only LCA affects IFNγ expression of Jurkat T cells (Fig 1K). To further characterize the inhibition of ERK phosphorylation by LCA, a time course experiment was performed. ERK1 and ERK2 are phosphorylated within 15 minutes in response to PMA/ionomycin, and remain elevated up to 180 minutes after activation (Fig 3C and 3D). LCA significantly decreases ERK1 and ERK2 phosphorylation at most time points tested (Fig 3C and 3D). These results suggest that LCA affects Th cell function via inhibition of ERK phosphorylation.

### LCA inhibits Th1 differentiation of CD4^+^ Th cells

**Fig 4:**
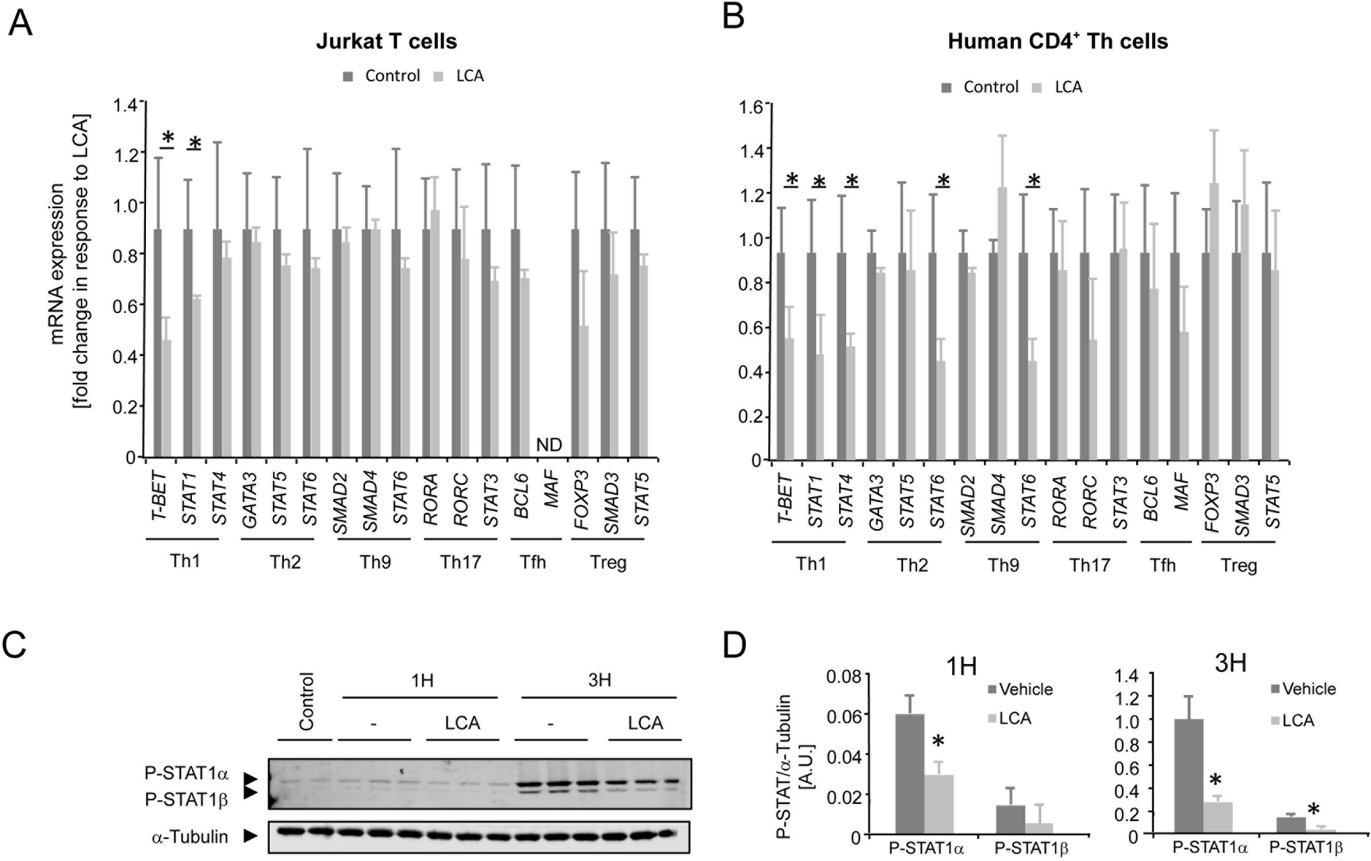
LCA inhibits the Th1 differentiation response of CD4^+^ Th cells. **(A)** mRNA expression in P/I-activated Jurkat T cells and **(B)** Dynabeads T-activator stimulated primary human CD4^+^ Th cells of a panel of genes associated with Th differentiation in response to 10 μM LCA treatment. ND; not detected. **(C)** Western blot images and **(D)** Quantification results of phosphorylated STAT1α/β and α-Tubulin in Jurkat T cells in response to P/I stimulation in combination with 10 μM LCA (light grey bars) or vehicle treatment (dark grey bars). LCA, lithocholic acid; P/I, PMA/ionomycin; ND, Not detected; AU, Arbitrary units. Results represent the mean ± SEM. *Statistically significant, P<0.05. Experiments were performed in triplicates and repeated at least twice.

Th cells can differentiate upon antigen exposure into several subsets of Th cells that have specific functions in immunity [10]. We observed a robust inhibition of ERK phosphorylation by LCA. Given that ERK signaling has been linked to Th differentiation [24], we next aimed to investigate whether the effect of LCA on CD4^+^ Th cells involves changes in differentiation of the cells. For this purpose we used Jurkat T cells, which upregulated both Th1-associated genes, such as *T-box transcription factor 21 (TBX21 also known as T-BET)* as well as Th2-associated genes, such as *GATA Binding Protein 3* (*GATA3*) in response to PMA/ionomycin stimulation (Panel A and B of S3 File). Jurkat T cells were treated with LCA in combination with PMA/ionomycin stimulation and we subsequently assessed a panel of genes that are associated to specific Th subsets, notably Th1, Th2, Th9, Th17, Tfh or T-regulatory cells. Interestingly, LCA specifically inhibits two genes that are linked to Th1 differentiation; *Signal transducer and activator of transcription (STAT)-1* and *T-BET*, whereas other tested genes are unaffected by LCA (Fig 4A). These data indicate that LCA inhibits Th cell cytokine production by blocking Th1 differentiation.

To confirm these findings in a more physiological setting, we stimulated primary human CD4^+^ Th cells with Dynabeads T-activator in combination with LCA treatment. In line with the findings in the Jurkat T cells, we observed inhibition of the Th1-associated genes *T-BET*, *STAT1* and *STAT4* (Fig 4B). Other genes tested were not altered upon LCA treatment, except for *STAT6*, which was inhibited by LCA (Fig 4B).

Phosphorylation of STAT1 at Tyr701 increases STAT1 dimerization, nuclear localization, DNA binding and is a key factor in Th1 commitment [25]. Additionally, STAT1 activation underpins expression of the Th1 gene T-BET [25], which we found decreased in Th cells upon LCA exposure. We therefore aimed to investigate STAT1 phosphorylation at Tyr701 in Jurkat T cells in response to LCA treatment. PMA/ionomycin treatment induced clear phosphorylation of STAT1α/β (Fig 4C and 4D). In line with the finding that LCA inhibited Th1-associated genes, a strong inhibition of phosphorylation of both STAT1α as well as STAT1β was observed (Fig 4C and 4D). Together with our finding that Th1-associated cytokines (IFNγ and TNFα) are downregulated by LCA, these data clearly indicate that LCA acts on CD4^+^ Th cells and impedes Th1 differentiation.

### Vitamin D receptor senses LCA in CD4^+^ Th cells

**Fig 5:**
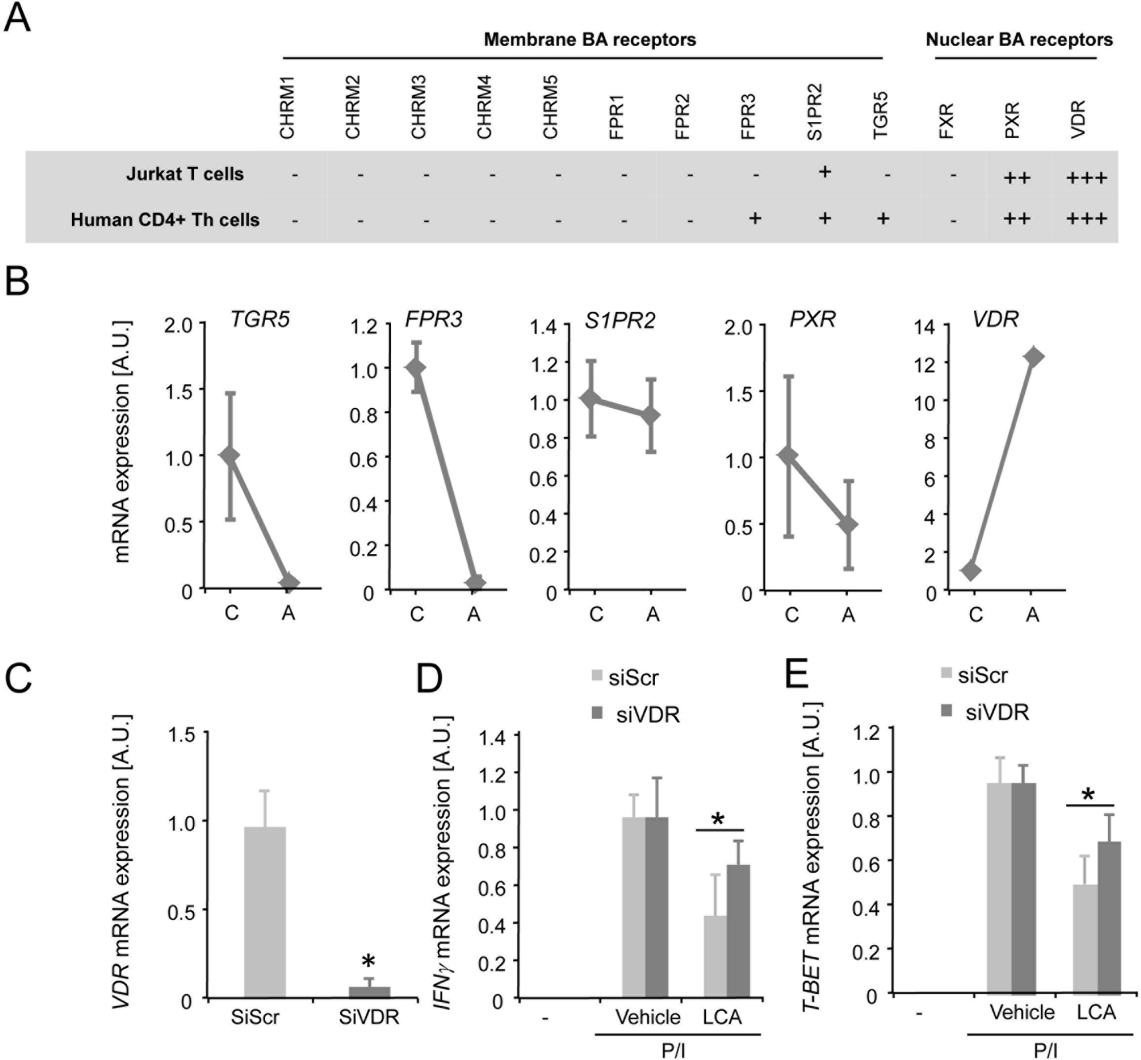
Characterizing the LCA sensor in CD4^+^ Th cells. **(A)** Table showing mRNA expression of Membrane and Nuclear bile acid receptors in Jurkat T cells and primary human CD4^+^ Th cells. “+” indicates that the gene is expressed at low levels, i.e. Real-time RT-PCR Ct cycles of 30–35; “++” indicates that the gene is expressed at Ct cycles of 25–30, and “+++” indicates high expression of the gene at Ct cycles of <25. **(B)** mRNA expression of *TGR5*, *FPR3*, *S1PR2 PXR* and *VDR* in resting “C” and in Dynabeads T-activator stimulated primary human CD4^+^ Th cells “A”. **(C)** mRNA expression of VDR in Jurkat T cells transfected with silencing RNA against the VDR (siVDR; light grey bar) or scrambled siRNA (siSCR; dark grey bar). **(D)** *IFN*γ and **(E)** *T-BET* mRNA expression in Jurkat T cells transfected with siRNA against the VDR (siVDR; light grey bars) or scrambled siRNA (siScr; dark grey bars) in response to P/I stimulation and in combination with 10 μM LCA or vehicle treatment. LCA, lithocholic acid; P/I, PMA/ionomycin; AU, Arbitrary units. Results represent the mean ± SEM. NS, Not significant. *Statistically significant, P<0.05. Experiments were performed in triplicates and repeated at least twice.

We next aimed to identify the LCA sensor in CD4^+^ Th cells to gain insight into the mechanism by which LCA controls Th1 differentiation. For this purpose, we analyzed expression of a number of nuclear and membrane receptors known to be involved in bile acid signaling in Jurkat T cells and primary human CD4^+^ Th cells [4]. We observed expression of the membrane receptors *TGR5*, *Formyl peptide receptor 3* (*FPR3*) and *Sphingosine-1-phosphate receptor 2* (*S1PR2*) and expression of the nuclear receptors *Vitamin D receptor* (*VDR*) and *Pregnane X receptor* (*PXR*) (Fig 5A). More detailed mRNA expression analysis revealed that *FPR3* and *TGR5* are greatly downregulated upon T cell activation, whereas mRNA expression of *VDR* is over 10 fold induced upon activation (Fig 5B).

Since TGR5 inhibits inflammation [4], and is expressed to some extend on CD4^+^ Th cells, we investigated whether TGR5 is involved in the inhibition of Th1 differentiation. For this purpose, Jurkat T cells were transfected with a TGR5 overexpressing plasmid by electroporation, which gives high transfection rates in Jurkat T cells as analyzed by fluorescent microscopy and flow cytometry (Panel C-F of S3 File). Transfection of Jurkat T cells with the TGR5 plasmid resulted in enhanced *TGR5* mRNA expression (Panel G of S3 File). TGR5 is activated in Jurkat T cells in response to LCA as measured by luciferase activity with cotransfection of a CREB reporter plasmid (Panel H of S3 File). However, we were unable to link TGR5 to the inhibitory action of LCA on Th1 differentiation as analyzed by mRNA expression of the key Th1 genes *IFN*γ and *T-BET* (Panel I and J of S3 File).

The *VDR* is expressed relatively high in CD4^+^ Th cells (Fig 5A and 5B) and binds to LCA [26]. We therefore investigated involvement of the VDR in mediating the effect of LCA on Th1 differentiation. Jurkat T cells were transfected with scrambled control RNA (control siRNA) and small interfering RNA against the VDR (siVDR). Transfection of Jurkat T cells with siVDR resulted in clear downregulation of *VDR* mRNA expression (Fig 5C). Interestingly, Jurkat T cells in which the VDR is silenced using siRNA are less responsive to LCA-mediated inhibition of the key Th1 genes *IFN*γ end *T-BET* (Fig 5D). Collectively, these data demonstrate that LCA signals predominantly but not exclusively via the VDR to inhibit Th1 differentiation of CD4^+^ Th cells.

## Discussion

Bile acids are appreciated as multifunctional molecules having roles that range from the emulsification of lipids to hormone-like signaling at the crossroad between food sensing and metabolism [4]. In the current study we screened common bile acid species to investigate the potency of bile acids with regard to their ability to affect adaptive immune cell responses to uncover novel signaling roles of bile acids. We are the first to demonstrate that physiological concentrations of LCA inhibit Th1 activation as measured in Jurkat T cells and primary human/mouse CD4^+^ Th cells in a VDR-dependent manner. These data establish a yet unrecognized bile acid-adaptive immunity axis.

LCA inhibits adaptive immune responses by impairing Th1 activation. This was observed in Jurkat T cells as well as in human primary CD4^+^ Th cells as measured by decreased mRNA expression of the Th1-associated genes *STAT1*, *STAT4* and *T-BET* [27]. In line with these data, the Th1-associated cytokines TNFα and IFNγ were also repressed which further confirmed decreased Th1 activation in response to LCA exposure [27]. Finally, STAT1-Tyr701 phosphorylation, crucial in Th1 differentiation [25], is also decreased upon LCA treatment. These data argue that LCA impedes Th1 differentiation and Th1 proinflammatory responses. LCA strongly inhibits ERK1/2 phosphorylation in activated Jurkat T cells, but not other MAPK pathways. Interestingly, ERK2 is pivotal in Th1 differentiation [24], and is also associated to regulate STAT1-Tyr701 phosphorylation in myeloid cells [28, 29]. The LCA-induced blocking of ERK phosphorylation could therefore provide an explanation for the impaired Th1 activation.

Next to a strong inhibition of ERK phosphorylation, LCA increases basal phosphorylation levels of P38 in Jurkat T cells. This effect is lost upon activation of the cells. P38 signaling in T cells was recently demonstrated to contribute to T cell senescence and as such could restrain chronic inflammation [30]. It is interesting to speculate that the P38 phosphorylation induced by LCA contributes to T cell senescence, which could potentially contribute to the anti-inflammatory effect in Th cells. It however remains to be investigated whether LCA induces T cell senescence.

We profiled the bile acid sensor in Th cells, and observed that multiple bile acid receptors are expressed in Th cells, notably TGR5, FPR, S1PR2, PXR and VDR. Interestingly, the bile acid sensor in Th cells changes upon activation of the cells as TGR5 and FPR3 are downregulated and VDR is upregulated upon activation. It should also be noted that PXR only responds to high LCA concentrations of 30–100μM [31], and is unlikely to play a role in the effects of LCA observed. We observed that LCA inhibits Th1 activation at least partially via VDR, as demonstrated by siRNA silencing experiments. The VDR is specifically activated by LCA [26], which provides an explanation for the observation that other bile acid species were unable to inhibit Th cell activation. The finding that unconjugated, but not conjugated LCA, is capable to modulate adaptive immune responses fits with the intranuclear localization of the VDR since conjugated bile acids are less capable to cross cellular membranes. Interestingly, the function of the VDR is well-studied in T cells and also coupled to inhibition of Th1 differentiation [15–18], which is in line with our finding that LCA impedes Th1 activation. The precise mechanism by which LCA regulates inhibition of ERK phosphorylation and activation of P38 phosphorylation are yet unknown. It is however, very well possible that these kinases are regulated by activation of the VDR by LCA, as the VDR analogue EB1089 gives a similar phosphorylation profile in B lymphocytes [32].

Although some studies revealed pro-inflammatory effects of bile acids in immune responses [33, 34], far most evidence points to inhibition of innate immune responses by bile acids and the bile acid-receptor TGR5 [6, 9, 35–42]. Of interest, it has been shown that bile acids are able to differentiate dendritic cells into IL-12 hypo-producing dendritic cells via the TGR5-cAMP pathway [43], which indirectly inhibits adaptive immune responses. In the current study, we are the first to demonstrate that LCA inhibits adaptive immune responses by a direct action on Th cells via the VDR.

Bile acids are well known for their presence within the entero-hepatic cycle. Bile acids can also reach high levels in the peripheral circulation, where concentrations up to 10 μM have been reported in healthy subjects after food intake [2, 3]. LCA is a secondary bile acid that is abundantly present in the portal vein but also found in the peripheral circulation mainly in its unconjugated form, where it can reach concentrations up to 0.5 μM [3]. Interestingly, we observed that LCA already impacts on Th cell inflammation at concentrations of 0.5 μM. This indicates that physiological concentrations of LCA in the peripheral circulation of healthy humans are sufficient to impact on adaptive immune responses.

Bile acids are interlinked to many facets important in atherosclerosis and inflammation. For example, bile acids are part of the cholesterol elimination route and are also heavily dependent on a healthy liver function. This makes the interpretation of correlations between bile acid levels and inflammatory diseases challenging. One small study has revealed a negative correlation between LCA levels and coronary artery disease [44]. Coronary artery disease is driven by atherosclerosis, a chronic inflammatory disease to which Th1-polarized T cells significantly contribute [13]. Our data could potentially indicate that LCA adds to the protection against coronary artery disease by blocking Th1 responses, although this correlation should be confirmed in other (larger) clinical trials. Several other implications of our results also deserve further study, including potential effects on T helper-driven macrophage activation and polarization [45].

LCA is a secondary bile acid and its formation depends on a small population of gut bacteria in the *Clostridium* genus that express 7α-dehydroxylase [46]. Consequently, antibiotics that affect the gut flora are demonstrated to impact on LCA formation [47]. It is therefore interesting to speculate that diets, antibiotics and pro/prebiotics could possibly modulate adaptive immune responses via the novel LCA-Th cell axis that we here describe.

In conclusion, we demonstrate in Th cells that LCA impedes Th1 activation via the VDR. This is the first time that bile acids are shown to directly impact on adaptive immune responses, an unrecognized action that further expands the hormone-like signaling power of bile acids. Our data may have important implications regarding drug and food strategies that impact on bile acid metabolism, as our data argue that these could potentially impact on adaptive immune responses via a yet unrecognized LCA-Th cell axis.

## Supporting information

S1 File First supporting figure. (PDF)

S2 File Second supporting figure. (PDF)

S3 File Third supporting figure. (PDF)

S1 Table Primers for real-time RT-PCR analysis. (PDF)
