# Supplementary material for: Lithocholic acid controls adaptive immune responses by inhibition of Th1 activation through the Vitamin D receptor
Source: PLoS One. 2017 May 11;12(5):e0176715. doi: 10.1371/journal.pone.0176715 (PMC5426628; doi:10.1371/journal.pone.0176715)
Supplement: S1 File — (PDF) [file pone.0176715.s001.pdf]

S1 Figure

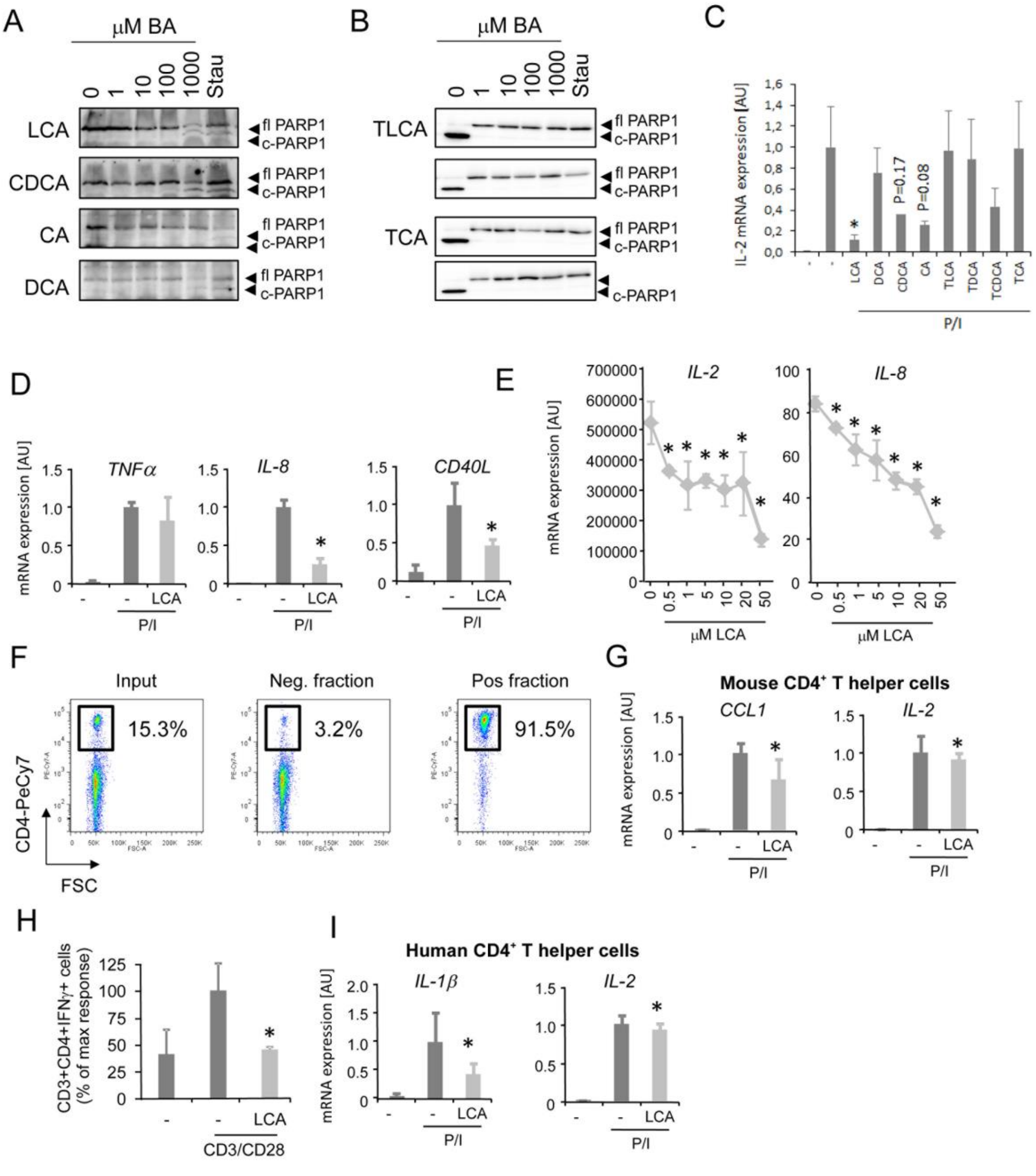

(A) Western blot images of cleaved PARP1 (c-PARP1) and full length PARP1 (fl PARP1) of Jurkat T cells treated for 24 hours with increasing concentrations of unconjugated bile acids and **(B)** taurine-conjugated bile acids. Stau; Staurosporine. **(C)** IL-2 mRNA expression of PMA/Ionomycin (P/I)-activated Jurkat T cells treated for 24 hours with 10 μM of different bile acid species. **(D)** mRNA expression of *TNF*α, *IL-8* and *CD40L* in Jurkat T cells activated with P/I and treated with 10 μM LCA (light grey bars) or vehicle (dark grey bars). **(E)** *IL-2* and *IL-8* mRNA expression in P/I-activated Jurkat T cells in response to increasing concentrations of LCA. **(F)** Flowcytometry plots of primary mouse $CD4^+$ T helper cells demonstrating successful isolation of $CD4^+$ T helper cells. **(G)** mRNA expression of *CCL1* and *IL-2* in primary mouse $CD4^+$ T helper cells activated with P/I and treated with 10 μM LCA (light grey bars) or vehicle (dark grey bars). **(H)** Intracellular IFNγ staining of primary mouse T helper cells isolated from spleen ($CD3^+CD4^+$) activated with CD3/CD28 and treated with 10 μM LCA (light grey bars) or vehicle (dark grey bars) analyzed by FACS. **(I)** mRNA expression of *IL-1β* and *IL-2* in primary human $CD4^+$ T helper cells activated with P/I and treated with 10 μM LCA (light grey bars) or vehicle (dark grey bars). LCA, lithocholic acid; CDCA, chenodeoxycholic acid; CA, cholic acid; DCA, deoxycholic acid; TLCA, taurolithocholic acid; TDCA, taurodeoxycholic acid; TCDCA, taurochenodeoxycholic acid; TCA, taurocholic acid; AU, Arbitrary units. P/I, PMA/ionomycin. Results represent the mean ± SEM. *Statistically significant, P<0.05. Experiments were performed in triplicates and repeated at least twice.
