# Supplementary material for: Lithocholic acid controls adaptive immune responses by inhibition of Th1 activation through the Vitamin D receptor
Source: PLoS One. 2017 May 11;12(5):e0176715. doi: 10.1371/journal.pone.0176715 (PMC5426628; doi:10.1371/journal.pone.0176715)
Supplement: S2 File — (PDF) [file pone.0176715.s002.pdf]

S2 Figure

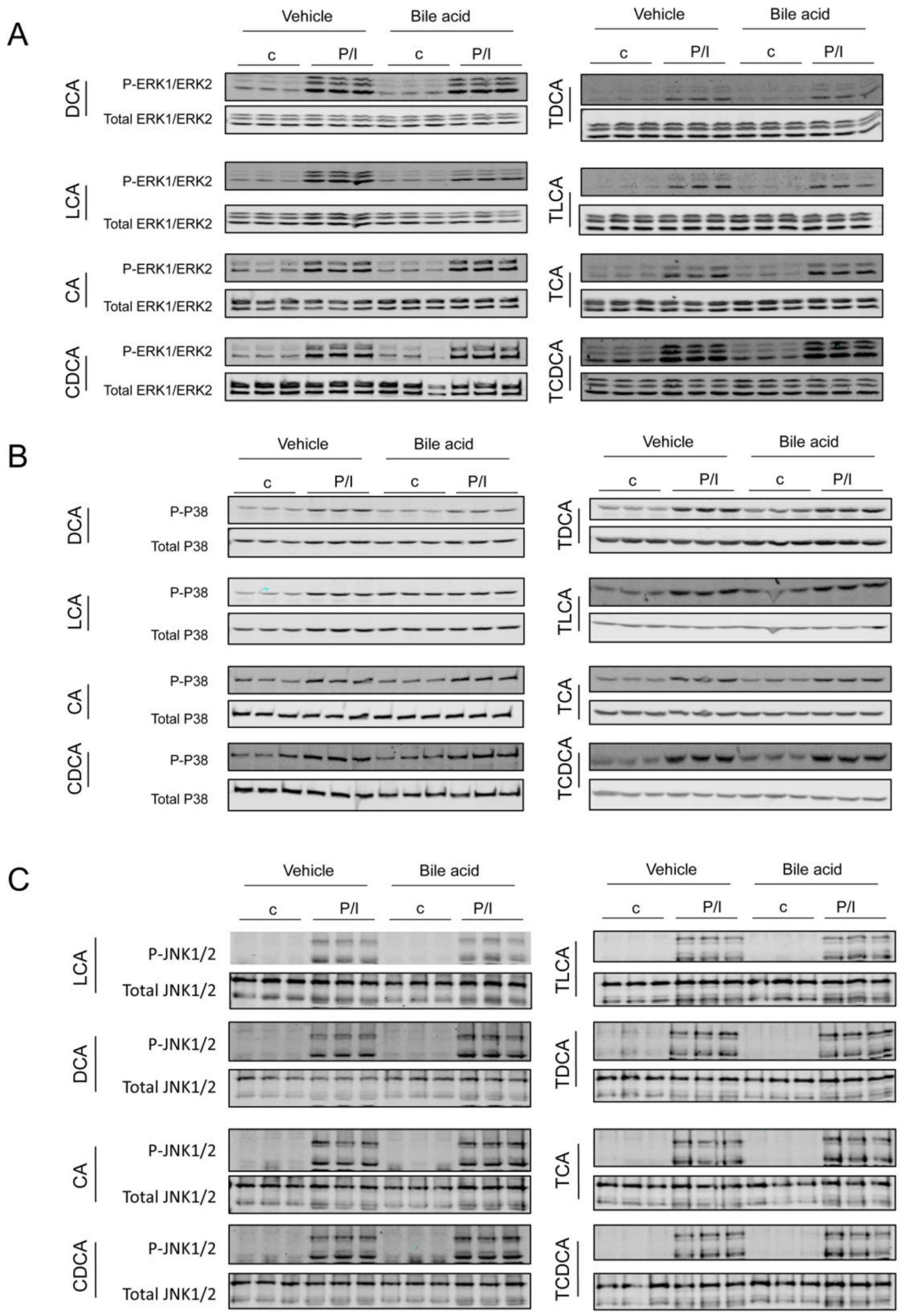

Western blot images of total and phosphorylated levels of **(A)** ERK1/2, **(B)** P38 and **(C)** JNK1/2 in response to 10 μM bile acid treatment or vehicle in P/I-activated or resting Jurkat T cells. LCA, lithocholic acid; CDCA, chenodeoxycholic acid; CA, cholic acid; DCA, deoxycholic acid; TLCA, taurolithocholic acid; TDCA, taurodeoxycholic acid, TCDCA, taurochenodeoxycholic acid, TCA, taurocholic acid. P/I, PMA/ionomycin.
