# Supplementary material for: Lithocholic acid controls adaptive immune responses by inhibition of Th1 activation through the Vitamin D receptor
Source: PLoS One. 2017 May 11;12(5):e0176715. doi: 10.1371/journal.pone.0176715 (PMC5426628; doi:10.1371/journal.pone.0176715)
Supplement: S3 File — (PDF) [file pone.0176715.s003.pdf]

S3 Figure

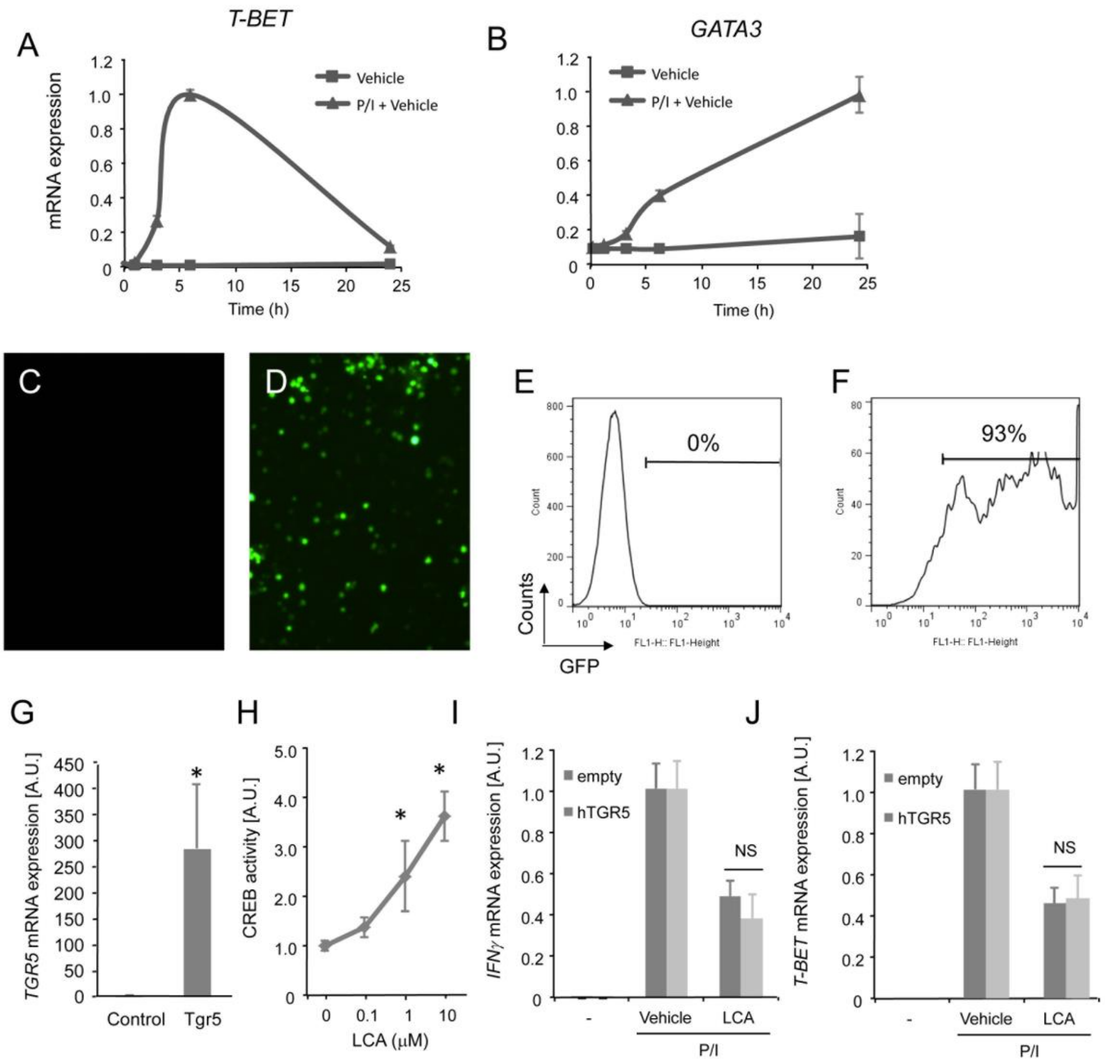

mRNA expression of **(A)** *T-BET* and **(B)** *GATA3* in response to P/I-activated Jurkat T cells (triangles) or in resting cells (squares). Results represent the mean ± SEM. P/I, PMA/ionomycin. **(C)** Pictures of fluorescent microscopy of Jurkat T cells transfected with control plasmids and **(D)** GFP-plasmids. **(E)** Flowcytometry analysis of Jurkat T cells transfected with control plasmids and **(F)** GFP-plasmids. **(G)** mRNA expression of *TGR5* in Jurkat T cells transfected with control plasmid and TGR5-overexpressing plasmids. **(H)** CREB activity as measured by luciferase activity in Jurkat T cells transfected with a plasmid encoding TGR5 and treated with increasing concentrations of LCA. **(I)** *IFN*γ and **(J)** *T-BET* mRNA expression of Jurkat T cells transfected with plasmids encoding TGR5 (hTGR5; light grey bars) or control plasmids (empty; dark grey bars) in response to P/I stimulation in combination with 10 μM LCA treatment. LCA, lithocholic acid. P/I, PMA/ionomycin. Results represent the mean ± SEM. NS, Not significant. *Statistically significant, $P<0.05$.
