# Supplementary material for: Lithocholic acid controls adaptive immune responses by inhibition of Th1 activation through the Vitamin D receptor
Source: PLoS One. 2017 May 11;12(5):e0176715. doi: 10.1371/journal.pone.0176715 (PMC5426628; doi:10.1371/journal.pone.0176715)
Supplement: S1 Table — (PDF) [file pone.0176715.s004.pdf]

Supporting Table 1: Primers for Real-time RT-PCR analysis

		Gene	Reference	Forward primer (5' to 3')	Reverse primer (5' to 3')
Mouse	Cytokines, chemokines, costimulatory molecules	*Cd40l*	NM_011616	CCTTGCTGAACTGTGAGGAGA	CTTCGCTTACAACGTGTGCT
		Tnf	NM_013693	ACGGCATGGATCTCAAAGAC	AGATAGCAAATCGGCTGACG
		IFNγ	NM_008337	CCAAGCGGCTGACTGAACTC	GCCTGTTACTACCTGACACATTCG
		Il-6	NM_031168	GTTCTCTGGGAAATCGTGGA	GGAAATTGGGGTAGGAAGGA
		Ccl1	NM_011329	GGCTGCCGTGTGGATACAG	AGGTGATTTTGAACCCACGTTT
		Il-2	NM_008366	TGAGCAGGATGGAGAATTACAGG	GTCCAAGTTCATCTTCTAGGCAC
	Housekeeping genes	*P0*	NM_007475	GGACCCGAGAAGACCTCCTT	GCACATCACTCAGAATTTCAATGG
		B2m	NM_009735	TTCTGGTGCTTGTCTCACTGA	CAGTATGTTCGGCTTCCCATTC
Human	Cytokines, chemokines, costimulatory molecules	*CD40LG*	NM_000074	GCTCCATTTATAGCCAGCCTC	GTTTGGCGGAACTGTGGGT
		TNFα	NM_000594	AGGACACCATGAGCACTGAAAG	AGGAGAGGCTGAGGAACAAG
		IFNγ	NM_000619	TCGGTAACTGACTTGAATGTCCA	TCGCTTCCCTGTTTTAGCTGC
		IL1β	NM_000576	TGGCAGAAAGGGAACAGAAAGG	GTGAGTAGGAGAGGTGAGAGAGG
		IL-2	NM_000586	AACTCCTGTCTTGCATTGCAC	GCTCCAGTTGTAGCTGTGTTT
		IL-6	NM_000600	CGCCTTCGGTCCAGTTG	TCGTTCTGAAGAGGTGAGTG
		IL-8	NM_000584	TGTTCCACTGTGCCTTGGTTTCTCC	TGCTTCCACATGTCCTCACAACATCAC
	Th-associated differentiation genes	*T-BET*	NM_013351	GTCCAACAATGTGACCCAGAT	ACCTCAACGATATGCAGCCG
		BCL6	NM_001130845	GGAGTCGAGACATCTTGACTGA	ATGAGGACCGTTTTATGGGCT
		FOXP3	NM_014009	GTGGCCCGGATGTGAGAAG	GGAGCCCTTGTCGGATGATG
		GATA3	NM_001002295	GCCCCTCATTAAGCCCAAG	TTGTGGTGGTCTGACAGTTCG
		MAF	NM_005360	CTGGCAATGAGCAACTCCGA	AGCCGGTCATCCAGTAGTAGT
		RORA	NM_134260	ACTCCTGTCCTCGTCAGAAGA	CATCCCTACGGCAAGGCATTT
		RORC	NM_005060	CTGGGCATGTCCCGAGATG	GAGGGGTCTTGACCACTGG
		SMAD2	NM_005901	CGTCCATCTTGCCATTCACG	CTCAAGCTCATCTAATCGTCCTG
		SMAD3	NM_001145102	TGGACGCAGGTTCTCCAAAC	CCGGCTCGCAGTAGGTAAC
		SMAD4	NM_005359	CTCATGTGATCTATGCCCGTC	AGGTGATACAACTCGTTCGTAGT
		STAT1	NM_007315	CGGCTGAATTTCGGCACCT	CAGTAACGATGAGAGGACCCT
		STAT3	NM_139276	ACCAGCAGTATAGCCGCTTC	GCCACAATCCGGGCAATCT
		STAT4	NM_001243835	GCTTAACAGCCTCGATTTCAAGA	GAGCATGGTGTTCATTAACAGGT
		STAT5	NM_003152	CGACGGGACCTTCTTGTTG	GTTCCGGGGAGTCAAACTTCC
		STAT6	NM_001178080	CGAGTAGGGGAGATCCACCTT	GCAGGAGTTTCTATCAAGCTGTG
	BA receptor genes	*CHRM1*	NM_000738	CTCTATACCACGTACCTGCTCA	CCGAGTCACGGAGAAGTAGC
		CHRM2	NM_000739	AACTCCTCTAACAATAGCCTGGC	GTTCCCGATAATGGTCACCAAA
		CHRM3	NM_000740	CACGTACCGAGCCAAACGAA	AGGACAAAGGAGATGACCCAA
		CHRM4	NM_000741	AGGACACTTCCAATGAGTCCA	TGTCTGCTTCGTCACAATCTG
		CHRM5	NM_012125	AGTCTGGCTTGTGACCTTTGG	TGTCAAGGGTCTTGTGATGGA
		FPR1	NM_002029	TGGGAGGACATTGGCCTTTC	GGATGCAGGACGCAAACAC
		FPR2	NM_001462	AGTCTGCTGGCTACACTGTTC	TGGTAATGTGGCCGTGAAAGA
		FPR3	NM_002030	GCTAGTCCACGGAGTCACCT	GGTAGGATGGCACTGAAAGAGA
		S1PR2	NM_004230	CATCGTCATCCTCTGTTGCG	GCCTGCCAGTAGATCGGAG
		TGR5	NM_001077191	CCCAGGCTATCTTCCCAGC	GCCAGGACTGAGAGGAGCA
		FXR	NM_001206978	GACTTTGGACCATGAAGACCAG	GCCCAGACGGAAGTTTCTTATT
		PXR	NM_022002	TTGCCCATCGAGGACCAGAT	GTCTCCGCGTTGAACACTGT
		VDR	NM_001017536	TCTCCAATCTGGATCTGAGTGAA	GGATGCTGTAACTGACCAGGT
	Housekeeping genes	*P0*	NM_001002	TCGACAATGGCAGCATCTAC	ATCCGTCTCCACAGACAAGG
		B2M	NM_004048	CTCGCGCTACTCTCTCTTTCT	TGCTCCACTTTTTCAATTCTCT
